# The proteasome as a target: How not tidying up can have toxic consequences for parasitic protozoa

**DOI:** 10.1073/pnas.1904694116

**Published:** 2019-05-13

**Authors:** Elizabeth A. Winzeler, Sabine Ottilie

**Affiliations:** ^a^Department of Pediatrics, University of California San Diego School of Medicine, La Jolla, CA 92093

With modern drug discovery technologies, there are opportunities to discover drugs that are uniquely suited for treatment of specific parasitic diseases and have few of the liabilities of older, historical medicines. In PNAS, Wyllie et al. (1) used state-of-the-art methods to identify a clinical candidate for leishmaniasis and to discover its mechanism of action.

Leishmaniasis is a neglected parasitic infectious disease that can have different clinical manifestations depending on the parasite species. Visceral leishmaniasis (VL) (also known as kala-azar or black fever), which is caused by *Leishmania donovani* and *Leishmania infantum*, is the most severe form and marked by a swollen spleen. It is a significant cause of morbidity in areas where the insect vector, the sandfly, is present and causes up to 20,000 to 65,000 annual deaths (ref. 2 and https://www.who.int/en/news-room/fact-sheets/detail/leishmaniasis). Treatments for VL are suboptimal and consist of a variety of highly toxic historical molecules that would likely not be licensed if developed today. In addition, many require long treatment times (up to 30 d), some require hospitalization, most are not orally bioavailable, and resistance to some is suspected. Miltefosine, a repurposed breast cancer medication, is one of the best treatment options but still requires long dosing regimens and shows variable efficacy against different parasites (reviewed in ref. 3). There is currently no vaccine.

With a goal of finding better treatment alternatives, Wyllie et al. (1) first took a set of compounds that was active against another parasite, *Trypanosoma cruzi*, the etiological agent of Chagas’ disease and a cousin of *Leishmania* spp., and then retested these compounds in a laborious, high-content-imaging intracellular macrophage replication assay against *L. donovani* (4), identifying a weakly active compound that showed promise. With several modifications, they were able to turn this screening hit into a compound that was suitable for target identification studies and, ultimately, into a clinical candidate (GSK3494245, also described as compound 8) that could be tested in a mouse model of VL. This orally bioavailable compound worked well in a mouse model of VL. Furthermore, GSK3494245 demonstrates a desirable safety profile, good pharmacokinetics, and is now being progressed toward human clinical trials.

In addition to its potential to radically improve the treatment options for VL, another noteworthy feature of the work is the elegant and thorough approach that was used to determine the mechanism of action of GSK3494245 in parasites. Suspecting that the mechanism of action might be shared across closely related parasites, Wyllie et al. (1) first tested a compound from the series (compound 7) against a genome-wide *Trypanosoma brucei* RNA interference (RNAi) library (5). This RNAi library consists of ∼750,000 clones, each transformed with one RNAi construct under the control of a tetracycline-inducible promoter and covers >99% of the ∼7,500 nonredundant *T. brucei* gene set (5). To identify parasite knockdown clones that showed increased resistance to the compound, the authors isolated DNA from the library before and after tetracycline induction in the presence of compound 7. They then created samples for next-generation sequencing by amplifying DNA fragments containing RNAi cassette-insert junctions in semispecific PCR reactions in a process called RNAi target sequencing (RIT-seq) (6). Sequencing these samples showed that some of the parasites that were resistant to compound 7 bore interfering RNAs that mapped to the protein degradation pathway.

Because resistance does not necessarily reveal a target (genetically knocking down a true target should theoretically render parasites more sensitive to an inhibitor, rather than resistant), Wyllie et al. (1) next created drug-resistant parasite lines using in vitro evolution. Because the interpretation of whole-genome sequencing data for *Leishmania* parasites is thought to be messier than for other parasites, and because of previous publications showing that the proteasome is a druggable target in *Leishmania* (7), Wyllie et al. examined the genome sequence of candidate genes in the proteasome pathway. Selective sequencing revealed that all resistant mutants bore homozygous mutations within the genes encoding the β4 and β5 subunits of the parasite proteasome. To confirm the proteasome as the target, the team next overexpressed the subunits and showed that overexpression conferred resistance, as did editing the point mutations into the genome.

Another recent scientific advance that has allowed the discovery of better treatments for neglected parasitic diseases has been the development of high-resolution cryoelectron microscopy (cryo-EM). This powerful method can be used to solve structures of macromolecular complexes such as the proteasome, allowing a detailed understanding of how compounds bind. To further confirm on-target activity, Wyllie et al. (1) used cryo-EM to show that compound 8 bound the *Leishmania tarentolae* 20S proteasome in a previously undiscovered inhibitor site that lies between the β4 and β5 proteasome subunits. The mutations suggested that GSK3494245 would inhibit the chymotrypsin-like activity of the β5 subunit, and this was confirmed in biochemical assays.

In PNAS, Wyllie et al. used state-of-the-art methods to identify a clinical candidate for leishmaniasis and to discover its mechanism of action.

Overall, the data reconfirm the importance of the proteasome as a target for many diseases. The proteasome is a multisubunit complex present in all eukaryotes and archaea. The eukaryotic ubiquitin proteasome system is responsible for the degradation of ∼80% of all cellular proteins in eukaryotes (reviewed in ref. 8). Proteasome inhibitors (PIs) first emerged as a powerful tool in the treatment of multiple myeloma, successfully leading to three Food and Drug Administration-approved drugs. While broadly acting (i.e., species-nonselective) PIs have long been known to have antiparasitic effects [e.g., against *Schistosoma* (9) and *Babesia* (10)], their action on host proteasomes precluded their development for the treatment of infectious disease. The first evidence for the ability of small chemical compounds to inhibit the proteasome of an infectious agent while sparing the proteasome of its host changed this picture (11). In addition to GSK3494245, species-selective PIs have been now identified for a variety of parasitic organisms such as *Plasmodium* (12–15), which are very sensitive to many classes of PIs. Despite its high level of conservation, the identification of compounds that appear nontoxic but are, nevertheless, able to kill various eukaryotic pathogens suggests that selectivity can be achieved and that the old dogma that pathogens cannot share targets with humans is untrue.

**Fig. 1.**
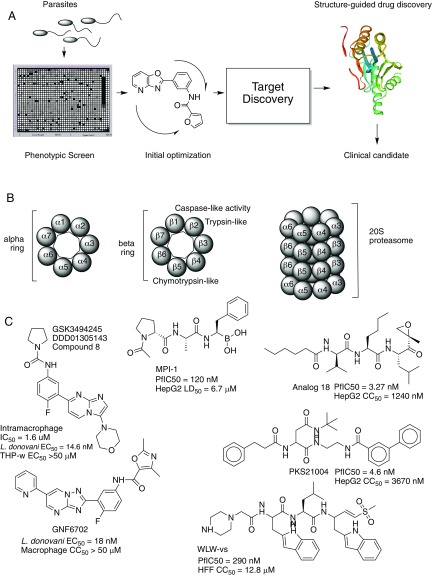
(*A*) Strategy for finding high-value treatments for neglected diseases. Compounds are first discovered using phenotypic screens, and active compounds are then subjected to several rounds of improvement. The target of active compounds is next identified using methods such as in vitro evolution and whole-genome analysis or using over-/underexpression libraries (5). Knowledge of the target can then lead to better leads with high specificity. (*B*) Diagram of the 20S proteasome subunit showing the position of β subunits that catalyze protein degradation and which are inhibited by GSK3494245 (compound 8). (*C*) Structures of various inhibitors that show specificity for parasite proteasomes over human ones. Compounds are described in detail elsewhere: GSK3494245 (1), HT1171 (11), MPI-1 (13), analog 18 (17), GNF6702 (7), PKS21004 (15), and WLW-vs (14). GNF6702 and GSK3494245 are active against *Leishmania* spp., while others act against the human malaria parasite *P. falciparum* or *Mycobacteria tuberculosis*. THP-w, HepG2s, macrophages, Vero76, and human foreskin fibroblasts (HFF) are mammalian cells used in toxicity tests. Amounts that give a 50% reduction in viability in whole-cell dose–response assays include effective concentration (EC_50_), lethal dose (LD_50_), and cytotoxicity concentration (CC_50_).

An open question is whether resistance will appear readily during treatment, given that Wyllie et al. (1) were able to create parasite lines that showed 100X resistance. It is also not entirely clear if the mutated genes would have been as easily identified if the proteasome were not a known target for trypanosomes (see refs. 7 and 16). On the other hand, work in *Plasmodium* spp. has shown that proteasome mutations can readily be discovered without prior knowledge using in vitro evolution and whole-genome analysis methods (13, 15), and the proteasome appears to be a high-value target for malaria as well (Fig. 1). Malaria PIs synergize with artemisinin derivatives, which are recommended for the treatment of malaria, and this could make them even more attractive candidates for drug development (14). One improvement between the compounds identified for VL and those which have activity in malaria parasites may be the cost of goods as well as oral bioavailability. Compound 8 can be made with fewer than six synthetic steps and should thus be affordable. Another interesting point is the structural differences between different PIs that have been discovered for different parasites (Fig. 1*C*). For example, GN6702 (7) and compound 8 are structurally different from known parasite PIs such as WLW-vs (14), asparagine ethylenediamines such as PKS21004 (15), and the carmaphycin B analog 18 (17).

Another drawback of other preclinical antiparasitic PIs as well as approved PIs for cancer treatment is their lack of good bioavailability. Ixazomib is the first and only oral PI and was recently approved for the treatment of multiple myeloma (18). Nevertheless, with knowledge of the structure, increasingly selective and potent compounds can likely be designed, and some of these may show efficacy in human trials. Wyllie et al.’s study shows that when all the pieces for drug discovery—including a chemically validated target, a structure, as well as biochemical and cellular assays—are in place, better treatments for neglected diseases can readily be found.
